# miR-135a inhibits airway inflammatory response in asthmatic mice via regulating JAK/STAT signaling pathway

**DOI:** 10.1590/1414-431X202010023

**Published:** 2021-01-15

**Authors:** Xue-peng Huang, Cheng-yu Qin, Yue-ming Gao

**Affiliations:** People's Hospital of Rizhao Lanshan, Respiratory Department, RizhaoShandong China Respiratory Department, People's Hospital of Rizhao Lanshan, Rizhao, Shandong, China

**Keywords:** miR-135a, JAK/STAT signaling pathway, Asthma, Airway inflammation

## Abstract

The objective of this study was to investigate the inhibitory effect of miR-135a in regulating JAK/STAT signaling pathway on airway inflammation in asthmatic mice. An asthma model was established by sensitization and stimulation with ovalbumin (OVA), and the corresponding drug intervention was given from the day of stimulation by means of nasal drops. Airway hyperresponsiveness was tested. The content of miR-135a in the lung tissue of mice was detected by RT-PCR. The pathological changes of lung tissue were evaluated by HE staining. Tumor necrosis factor (TNF)-α, interleukin (IL)-6, IL-5, and eotaxin in bronchoalveolar lavage fluid (BALF) and lung tissue were detected by ELISA and immunohistochemistry, respectively. The expression of JAK/STAT signaling pathway-related protein in lung tissue was detected by western blot. To further validate the effect of miR-135a overexpression on the JAK/STAT signaling pathway, pathway activators and inhibitors were added. Compared with the OVA group, the airway hyperresponsiveness of the mice was significantly decreased after treatment with the miR-135a agonist. The expression of miR-135a was significantly increased in the lung tissue and the pathological changes of the lung tissue were alleviated. The contents of TNF-α, IL-6, IL-5, and eotaxin in BALF and lung tissues were decreased. The expression of JAK/STAT signaling pathway-related proteins p-JAK3/JAK3, p-STAT1/STAT1, and p-STAT3/STAT3 were significantly reduced in lung tissue (P<0.05). Addition of JAK inhibitor AG490 reduced airway inflammation in asthmatic mice. miR-135a agonists inhibit airway inflammation in asthmatic mice by regulating the JAK/STAT signaling pathway.

## Introduction

Asthma is a complex inflammatory disease that is prone to long-term recurrence. It affects about 3 million people worldwide from infants to old age, and can cause 0.250-0.345 million deaths per year (1,2). Asthma has the following characteristics: reversible airflow obstruction, airway hyperresponsiveness, airway inflammation, inflammatory cell infiltration, mucus secretion, and bronchospasm (3,4). However, the potential pathogenesis and molecular and cellular mechanisms of the disease are still unclear, and the current treatment is only limited to the treatment of asthma symptoms and there is no complete cure (5,6). Therefore, it is of great clinical significance to seek an effective treatment for asthma (7).

Asthma is a common respiratory allergic disease, which seriously endangers human health, and its incidence is increasing yearly (8). The main pathological changes are the disorder of overall and local immune inflammatory responses, especially the non-specific airway inflammation mainly caused by eosinophils, mast cells, and their secreted inflammatory mediators and cytokines (9,10). The 2009 guidelines for the prevention and treatment of asthma (11) first proposed the concept of “phenotype” and that phenotypic classification could be helpful for the prognosis and treatment of asthma. Common asthma phenotypes include inflammatory phenotypes, clinical phenotypes, age-related phenotypes, trigger phenotypes, therapeutic response phenotypes, and obesity-related phenotypes (12). The pathogenesis of asthma can now be summarized as the result of airway immune inflammatory mechanisms, neuromodulation mechanisms, and the interaction of polygenic inheritance and environment (13). Research has shown that the onset of asthma is highly correlated with polygenic inheritance, and the role of genetic factors in the pathogenesis of asthma may be related to susceptibility genes (14). The main treatment methods are traditional medicine, targeted therapy, and interventional therapy (15).

The traditional treatment for asthma is symptoms control. Commonly used drugs are β2 adrenergic receptor agonist, M-choline receptor blocker, phosphodiesterase inhibitor, adrenocortical hormone, anti-leukotriene, etc (16). Two of these drugs are most frequently used, one is a short-acting selective β2 adrenergic agonist (SABA), which is a first-line treatment for acute asthma symptoms, and the other is a corticosteroid and a long-acting selective β2 adrenergic agonist (LABA). These are the most effective drugs for long-term control of asthma (17). However, some studies have shown that the use of corticosteroids does not completely control patients with severe asthma, and can have serious side effects. Therefore, the use of complementary and alternative drugs for asthma has become common (18). There are other treatments for asthma, such as Chinese medicine therapy, calcium antagonists, immunotherapy, specific antibodies, and gene therapy (19). In short, as the understanding of the pathogenesis of asthma continues to deepen, the treatment of asthma gets more diversified (20).

## Material and Methods

### Experimental animals

Fifty-four SPF Balb/c male mice (18-20 g, 6-8 weeks old) were purchased from Beijing Weitong Lihua Experimental Animal Technology Co., Ltd., license number SCXK 20160006 (China). The mice were kept in a sterile independent air supply isolation cage in an air laminar flow purification room, at a constant temperature (26-28°C), constant humidity (relative humidity 40-60%). The feed, drinking water, and litter were sterilized. All experiments followed the NIH guidelines (NIH Pub. No. 85-23, revised 1996) and have been reviewed by the Animal Protection and Use Committee of the People's Hospital of Rizhao Lanshan.

### Animal grouping

Twenty-four mice were randomly divided into 4 groups: normal control group (Control), model group (ovalbumin - OVA), miR-135a agonist (Guangzhou Ruibo Biotechnology Co., Ltd., China) group (miR-135a), and miR-135a agonist negative control group (Scramble), with 6 in each group. The miR-135a agonist sequence is: 5′-UAMGGCUUUUUAUUCCUAMGMGA-3′, 3′-AUACCGAAAAAUAAGGAUACACU-5′; the agonist negative control sequence is: 5′-UUMGUACUACACAAAAGUACMG-3′, 3′-AAACAMGAMGMGUUUUCAMGAC-5′.

### Mouse asthma model construction

Except for the control group, the other three groups were intraperitoneally injected with 50 μg of OVA (200 μL, containing 20 μg of ovalbumin and 2 mg of aluminum hydroxide; Sigma, USA) on the first and the 14th day of the experiment. From the 21st day of the experiment, 4% OVA solution was atomized and inhaled for 30 min/day for 1 week. For the control mice, the OVA sensitizing solution and the stimulating solution were replaced with the same amount of normal saline, and the other treatment methods were the same as the asthma group. In the miR-135a agonist group and the miR-135a agonist-negative control group, 20 μg of miR-135a agonist and miR-135a agonist negative control were intranasally injected 1 h before nebulization, 1 day before the experiment, 1 time/day, for a total of 7 times.

### Airway hyperresponsiveness test

The airway hyperresponsiveness test was performed after the last atomization for 24 h, and the spontaneous breathing of the mice was detected by plethysmography in the awake state. The mice to be tested were aerosolized with methacholine at different concentrations (0, 6.25, 12.5, 25, and 100 mg/mL) for 3 min, and the readings were recorded for 3 min and averaged. The results are reported as a percentage increase in airway resistance (Penh%) after nebulization with different concentrations of methacholine.

### Sample collection

After the airway hyperresponsiveness was detected, mice were intraperitoneally injected with 0.6% pentobarbital sodium (40 mg/kg), and intubated and fixed after anesthesia. Bronchopulmonary lavage was performed with ice-cold PBS, 0.8 mL/time for 3 times. The bronchopulmonary lavage fluid (BALF) was collected and centrifuged at 300 *g* for 5 min at 4°C. The supernatant was collected and stored at low temperature for use. After the mice were euthanized by cervical dislocation, the lung tissues of the mice were removed, and the blood on the lung surface was washed with ice-cold PBS buffer after sterilization. The left lung was placed in a 10% neutral formalin solution for 24 h, and the right lung was placed in an ultra-low temperature freezer.

### RT-PCR

Lung tissue samples were centrifuged (12,000 *g* for 10 min at 4°C) after grinding at 4°C using TRIzol (15596018, Invitrogen, USA) to extract total RNA (OD260/OD280 between 1.8 and 2.0 indicating acceptable RNA purity). The reverse transcription of microRNA was performed using the miScript Reverse Transcription kit (Qiagen, Germany). qRT-PCR was performed using a Mastercycler^®^ nexus X2 (Eppendorf, Germany). The conditions were: 94°C for 5 min, 94°C for 45 s, 59°C for 60 s, and 72°C for 90 s (35 cycles). The experimental data were processed by the 2^-ΔΔCt^ method and using U6 as the internal control to calculate the relative expression level. Primer (Shanghai Shenggong Bioengineering Technology Service Co., Ltd., China) sequences are as follows: miR-135a: forward: 5′-AACCCTGCTCGCAGTATTTGAG-3′, reverse: 5′-GCGGCAGTATGGCTTTTTATTCC-3′; U6: forward: 5′-GACCTCTATGCCAACACAGT-3′, reverse: 5′-AGTACTTGCGCTCAGGAGGA-3′.

### HE staining

The fixed lung tissue was paraffin-embedded and sliced (5 μm). The sections were dewaxed and hydrated. Sections were stained with hematoxylin (Solarbio Life Sciences, China) for 5 min and differentiated with hydrochloric acid ethanol for 30 s, and placed in eosin dye (Solarbio Life Sciences) for 2 min. The slices were dehydrated, stained, and covered.

### ELISA

The levels of tumor necrosis factor (TNF)-α (orb315038), interleukin (IL)-6 (orb79057), IL-5 (orb315047), and eotaxin (orb79001) (all from Biorbyt, UK) in the supernatant were detected in strict accordance with the ELISA kit instructions, and the data were read at 450 nm in a microplate reader (RT-6100, Rayto, China).

### Immunohistochemical staining

Paraffin blocks was routinely sectioned and dewaxed with xylene, and then successively hydrated with gradient ethanol solution. The solution was inactivated with 3% H_2_O_2_ methanol solution for 20 min, high-temperature antigen in citrate buffer (pH6.0) for 10 min, and sealed with 5% BSA for 20 min. Rabbit anti-mouse TNF-α (1:200, orb95065), IL-6 (1:50, orb228461), IL-5 (1:200, orb310603), and eotaxin (1:200, orb10625) polyclonal antibodies (all from Biorbyt) were added and reacted overnight at 4°C. After reheating, horseradish peroxidase-labeled goat anti-rabbit IgG (1:1000, ABIN101988, Antibodies-Online, Germany) was incubated with secondary antibodies, developed by DAB, re-stained, dehydrated, cleared, and sealed. The Aperio ImageScope 11.1 software (Leica Biosystems, USA) was used to count the results, which are reported as the percentage of positive cells (%).

### Western blot

The lung tissue samples were mechanically dispersed. After centrifugation (12,000 *g* for 10 min at 4°C, 10 min), the supernatant was removed and the BCA kit (Solarbio Life Sciences) was used to measure the protein concentration. A protein sample (40 μg) was mixed with 10% SDS gel buffer in a ratio of 1:1, and the protein was denatured by heating at 95°C for 5 min. The PVDF membrane was treated (Merck, Germany) at 80 V for 30 min. The membrane was blocked with 5% skim milk powder in Tris-buffered saline with Tween 20 (TBST) solution (Solarbio Life Sciences) for 1 h at 4°C, and the polyclonal antibodies of rabbit anti-mouse p-JAK3 (1:500, orb193083), JAK3 (1:500, orb161516), p-STAT1 (1:500, orb11426), STAT1 (1:500, orb223197), p-STAT3 (1:500, orb14778), STAT3 (1:500, orb99433), and β-actin (1:2000, orb178392) (all from Biorbyt) were diluted with TBST solution containing 3% bovine serum albumin. The reaction was carried out at 4°C overnight. After reheating, horseradish peroxidase-labeled goat anti-rabbit IgG (1:1000, ABIN101988, Antibodies-Online) was incubated for 1 h. After washing, ECL luminescent substrates were used for color development for 3-5 min. The protein levels were normalized to those of β-actin, and grayscale scanning and quantification were performed using ImageJ 1.8 software (NIH, USA).

### Animal grouping and administration

To further verify the effect of miR-135a overexpression on the JAK/STAT signaling pathway, a pathway activator (BDNF, Promega, USA) and an inhibitor (AG490, Sigma Aldrich, USA) were added. The experiment was divided into normal control group (Control), model group (OVA), miR-135a agonist group (miR-135a), JAK inhibitor group (AG490), and miR-135a agonist+JAK activator group (miR+BDNF), with 6 mice per group. On the 21st day of the experiment, the JAK inhibitor group was intraperitoneally injected with 10 mL/kg AG490 1 h before nebulization at a concentration of 100 μmol/L (μM) once daily for 7 days. In the miR-135a agonist+JAK activator group, 20 μg of miR-135a agonist was intranasally instilled 1 h before nebulization, followed by intraperitoneal injection of 3 μg/kg BDNF once daily for 7 days. Analyses with the above indicators were performed.

### Statistical analysis

SPSS 19.0 statistical analysis software (IBM, USA) was used for data processing, and the data analysis results are reported as means±SD. Univariate analysis of variance (ANOVA) was used for multi-group data analysis, and LSD test was used for subsequent analyses. P<0.05 indicated that the difference was statistically significant.

## Results

### Expression of miR-135a in mouse lung tissue and its effect on airway hyperresponsiveness in mice

**Figure 1:**
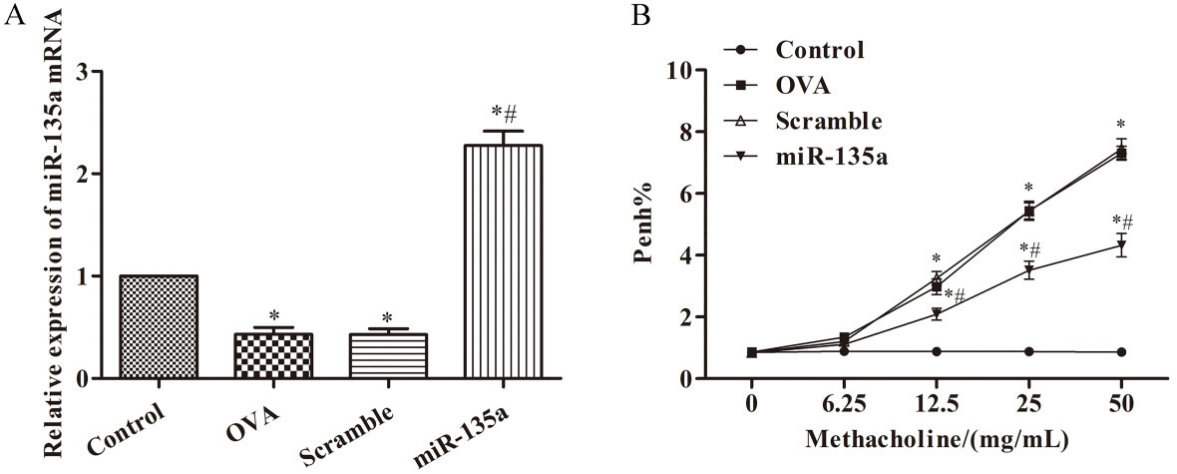
Expression of miR-135a in mouse lung tissue and its effect on airway hyperresponsiveness in mice. **A**, RT-PCR was used to detect the content of miR-135a mRNA in mouse lung tissue of different groups; (**B**), effect of miR-135a on airway hyperresponsiveness (Penh%) in mice of different groups. Data are reported as means±SD. *P<0.05 compared to the control group; ^#^P<0.05 compared to the ovalbumin (OVA) group (ANOVA).

As shown in Figure 1, the expression of miR-135a in the lung tissue of the OVA group was decreased compared with the control group (P<0.05). After nasal administration of miR-135a agonist, the expression of miR-135a in lung tissue of mice was significantly increased (P<0.05). With the increase in the concentration of methacholine, the airway hyperresponsiveness of the OVA group was increased compared with the control group, especially at the concentrations of 12.5, 25, and 50 mg/mL (P<0.05). Compared with the control group, the airway hyperresponsiveness of the miR-135a group was weakened (P<0.05), but there was no significant change in the Scramble group (P>0.05). From this, we can conclude that miR-135a was highly expressed in wheezing mice and can reduce airway hyperresponsiveness in mice.

### Effect of miR-135a on lung histopathology in mice

**Figure 2:**
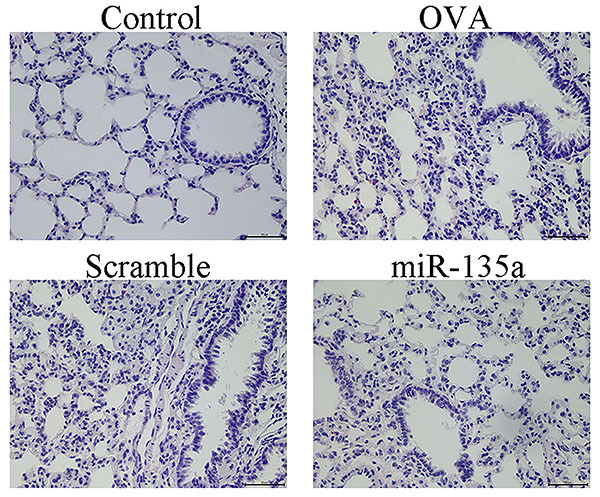
Effect of miR-135a on lung histopathology by HE staining in mice (×400, scale bar: 50 μm).

As shown in Figure 2, the mouse lung tissue structure in the control group was organized, the bronchial and alveolar wall structures were intact, there was no inflammatory cell infiltration around the bronchi, and no goblet cell hyperplasia. Compared with the control group, the mice in the OVA group had disordered lung tissue structure, significantly thickened alveolar walls, increased inflammatory cell infiltration around the bronchi, and significantly increased mucus secretion and goblet cell proliferation. Compared with the OVA group, the lung structure of the mice in miR-135a group was significantly improved, mucus secretion and the number of goblet cells were decreased, and inflammatory cells around the bronchi were also significantly reduced. The lung morphology of the Scramble group did not change significantly compared to the OVA group. The above experimental results showed that miR-135a can improve the pathological structure of lung tissue in mice.

### Effect of miR-135a on the expression of TNF-α, IL-6, IL-5, and eotaxin in lung tissue of mice

**Figure 3:**
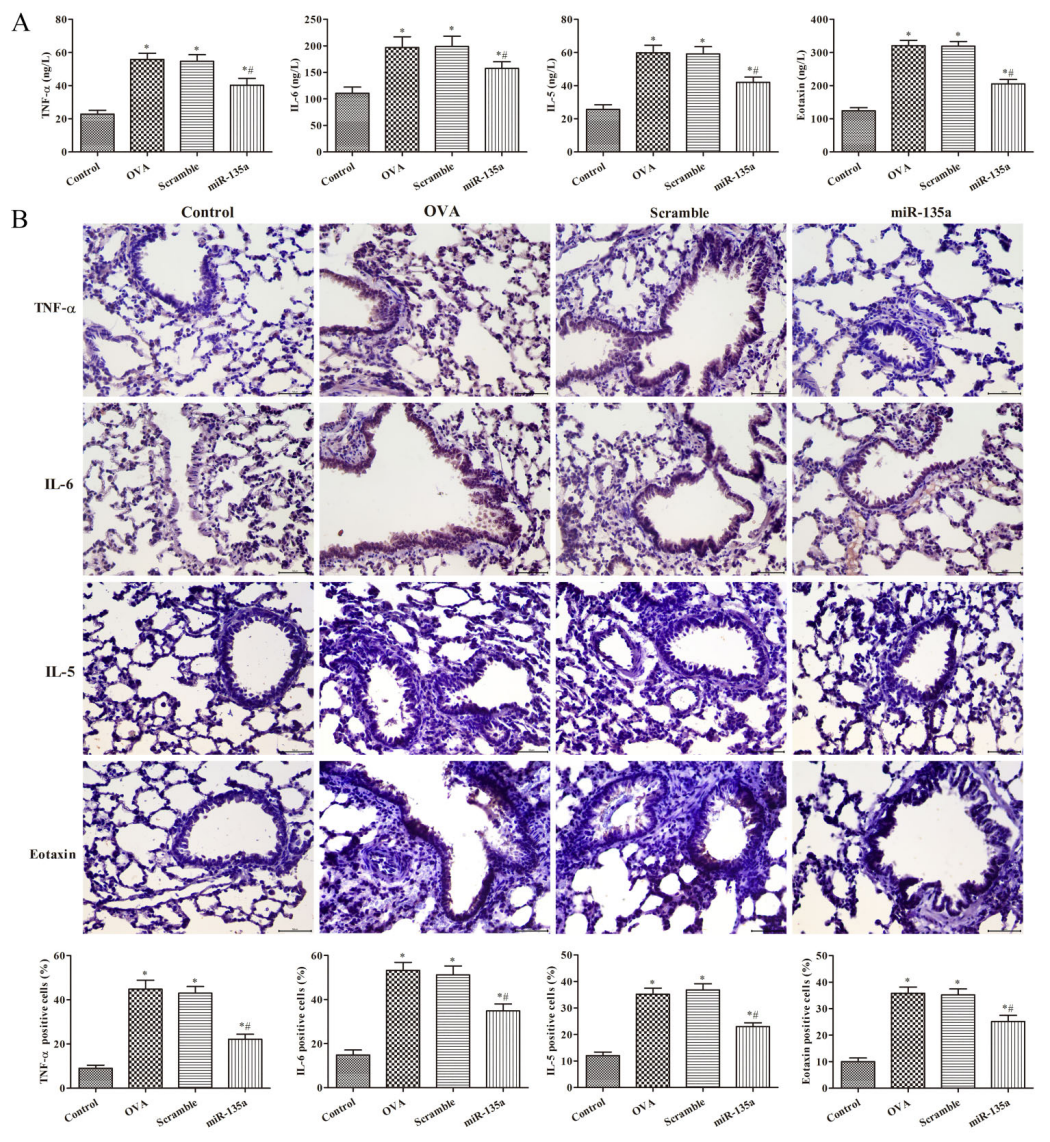
Effect of miR-135a on the expression of tumor necrosis factor (TNF)-α, interleukin (IL)-6, IL-5, and eotaxin in lung tissue of mice. **A**, ELISA was used to detect the content of TNF-α, IL-6, IL-5, and eotaxin in bronchoalveolar lavage fluid, and **B**, immunohistochemistry was used to detect the proteins in lung tissue of mice (×400, scale bar: 50 μm). Data are reported as means±SD. *P<0.05 compared to the control group; ^#^P<0.05 compared to the ovalbumin (OVA) group (ANOVA).

As shown in Figure 3A, compared with the control group, the levels of TNF-α, IL-6, IL-5, and eotaxin in the lung tissue of the OVA group were significantly increased (P<0.05). Compared with the OVA group, the levels of TNF-α, IL-6, IL-5, and eotaxin in the miR-135a group were significantly decreased (P<0.05), while not significantly changed in the Scramble group (P>0.05). As shown in Figure 3B, TNF-α was expressed in the cytoplasm and in some cell membranes. IL-6, IL-5, and eotaxin were expressed in cytoplasm. Compared with the control group, the levels of TNF-α, IL-6, IL-5, and eotaxin in the lung tissue of the OVA group were significantly increased (P<0.05). These levels in the miR-135a group were significantly decreased (P<0.05) compared to the OVA group, while they were not significantly changed in the Scramble group (P>0.05). miR-135a can reduce the levels of the inflammatory factors TNF-α, IL-6, IL-5, and eotaxin in mouse lung tissue, thereby reducing inflammation.

### Western blot analysis of JAK/STAT signaling pathway-related protein expression in mouse lung tissue

**Figure 4:**
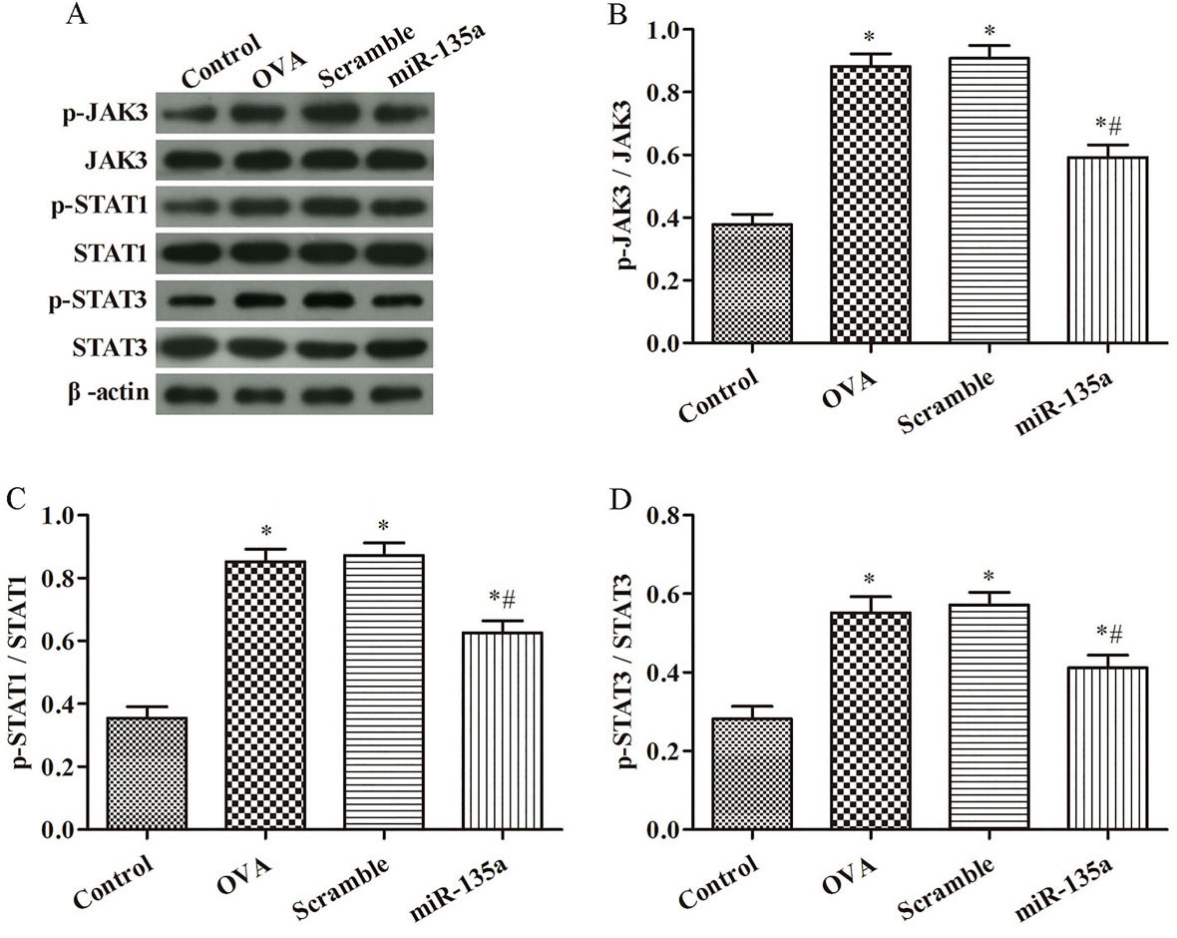
Western blot analysis of JAK/STAT signaling pathway-related protein expression in mouse lung tissue. **A**, Protein band diagram; **B**, p-JAK3/JAK3; **C**, p-STAT1/STAT1; **D**, p-STAT3/STAT3. Data are reported as means±SD. *P<0.05 compared to the control group; ^#^P<0.05 compared to the ovalbumin (OVA) group (ANOVA).

As shown in Figure 4, the levels of p-JAK3/JAK3, p-STAT1/STAT1, and p-STAT3/STAT3 proteins in the lung tissues of the OVA group were higher than those in the control group (P<0.05). Compared with the OVA group, p-JAK3/JAK3, p-STAT1/STAT1, and p-STAT3/STAT3 in the miR-135a group were significantly decreased (P<0.05), while in the Scramble group, they were not significantly changed (P>0.05).

### Effect of regulation of JAK/STAT signaling pathway on airway hyperresponsiveness in mice

**Figure 5:**
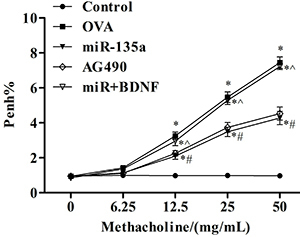
Effect of regulation of JAK/STAT signaling pathway on airway hyperresponsiveness (Penh%) in mice. Data are reported as means±SD. *P<0.05 compared to the control group; ^#^P<0.05 compared to the ovalbumin (OVA) group; ˆP<0.05 compared to the miR-135a group (ANOVA).

Figure 5 shows that the airway hyperresponsiveness of the miR-135a and AG490 mice was attenuated (P<0.05) compared with the OVA group as the concentration of methacholine increased. The airway hyperresponsiveness of the miR+BDNF group was enhanced (P<0.05) compared with the miR-135a group.

### Effect of regulation of JAK/STAT signaling pathway on pathology of lung tissue in mice

**Figure 6:**
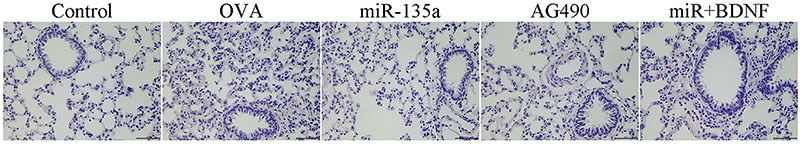
Effect of regulation of JAK/STAT signaling pathway on lung histopathology by immunohistochemistry staining in mice (×400, scale bar: 50 μm).

As shown in Figure 6, the mouse lung tissue structure in the control group was clear, the bronchial and alveolar wall structures were intact, there was no inflammatory cell infiltration around the bronchi, and no goblet cell hyperplasia. The mice in OVA group had a disordered lung tissue structure, a thickened alveolar wall, increased inflammatory cell infiltration around the bronchi, and increased mucus secretion and goblet cell hyperplasia. The lung structure of the miR-135a and AG490 mice was significantly improved, the mucus secretion and the number of goblet cells were reduced, and the inflammatory cells around the bronchi were also significantly reduced compared with the OVA group. Compared to the miR-135a group, pathological changes in lung tissue of mice in the miR+BDNF group were aggravated.

### Effect of regulation of JAK/STAT signaling pathway on the expression of TNF-α, IL-6, IL-5, and eotaxin in lung tissue of mice

**Figure 7:**
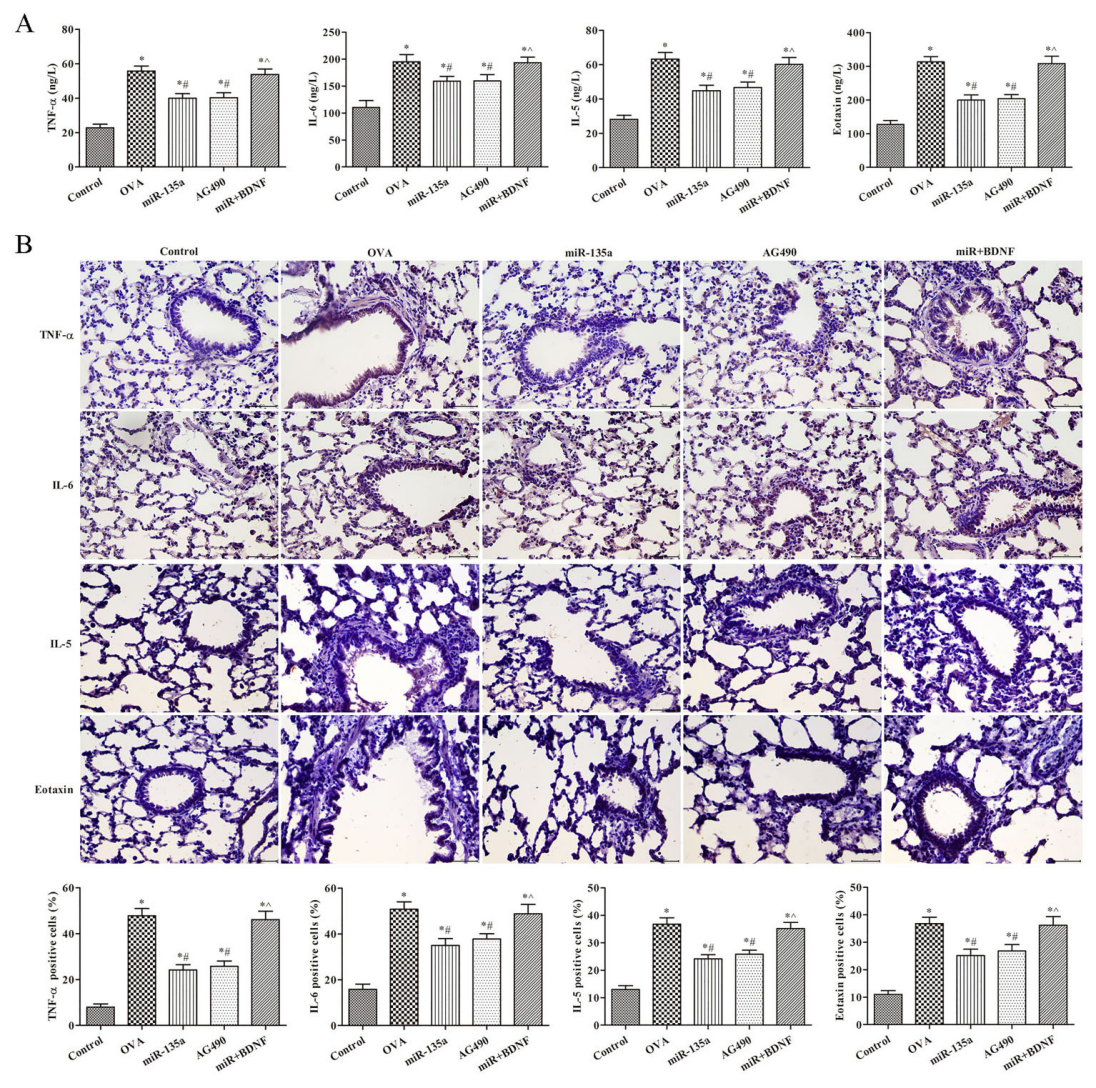
Effect of JAK/STAT signaling pathway on the expression of tumor necrosis factor (TNF)-α, interleukin (IL)-6, IL-5, and eotaxin in lung tissue of mice. **A**, The levels of TNF-α, IL-6, IL-5, and eotaxin in bronchoalveolar lavage fluid were detected by ELISA, and **B**, in lung tissue of mice by immunohistochemistry (×400). Data are reported as means±SD. *P<0.05 compared to the control group; ^#^P<0.05 compared to the ovalbumin (OVA) group; ˆP<0.05 compared with the miR-135a group (ANOVA).

As shown in Figure 7A and B, the levels of TNF-α, IL-6, IL-5, and eotaxin in the miR-135a and AG490 groups were lower than those in the OVA group (P<0.05); those of the miR-135a group were lower than those in the miR+BDNF group (P<0.05). Compared to the miR-135a group, the levels of TNF-α, IL-6, IL-5, and eotaxin in the miR+BDNF group were significantly increased (P<0.05). These results indicated that overexpression of miR-135a can inhibit JAK/STAT signaling pathway, and inhibition of this pathway can attenuate airway hyperresponsiveness, improve pathological tissue structure, and reduce the expression of inflammatory factors.

### Effect of regulation of JAK/STAT signaling pathway on the expression of pathway-associated proteins in mouse lung tissue

**Figure 8:**
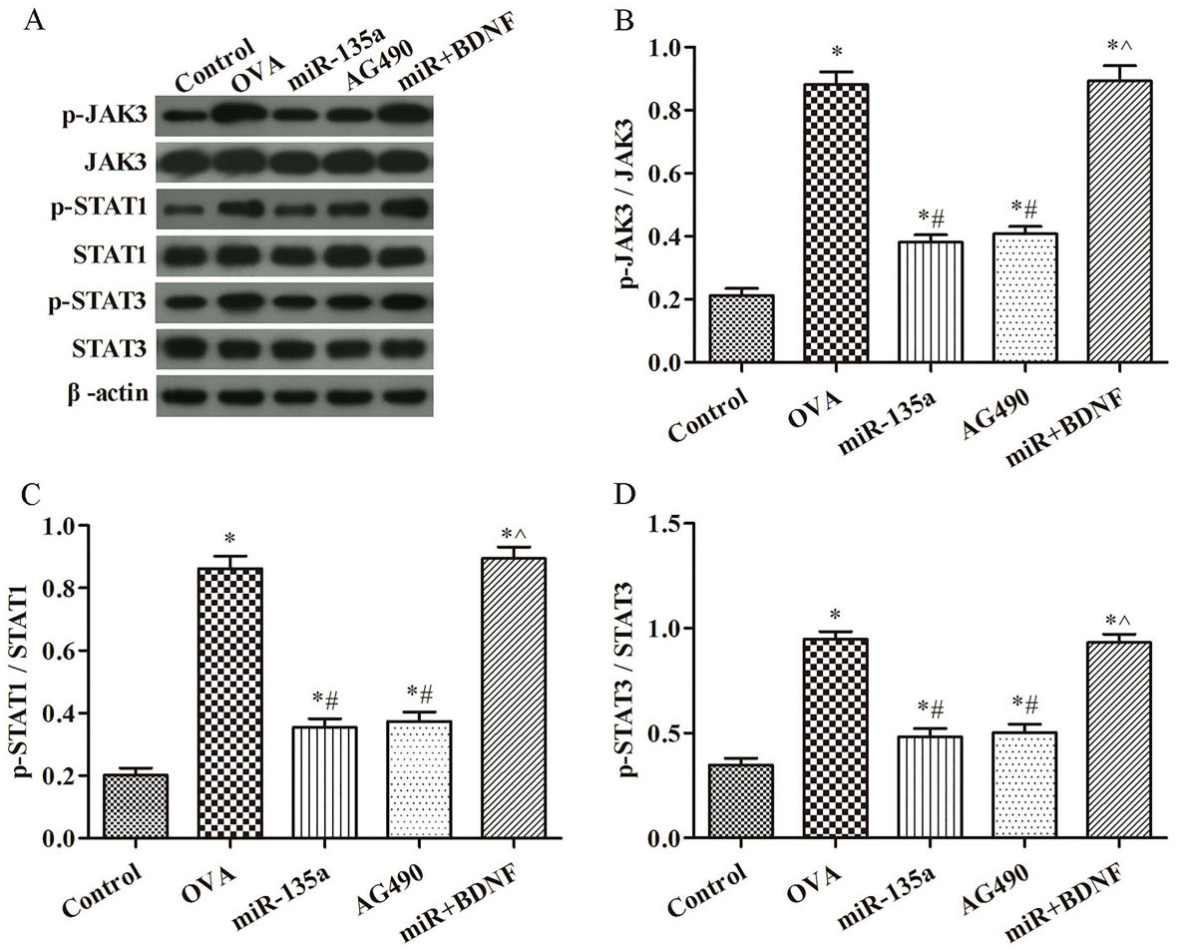
Effect of regulation of JAK/STAT signaling pathway on expression of pathway-associated proteins in mouse lung tissue. **A**, Protein band diagram; **B**, p-JAK3/JAK3; **C**, p-STAT1/STAT1; **D**, p-STAT3/STAT3. Data are reported as means±SD. *P<0.05 compared to the control group; ^#^P<0.05 compared to the ovalbumin (OVA) group; ˆP<0.05 compared to the miR-135a (ANOVA).

As shown in Figure 8, p-JAK3/JAK3, p-STAT1/STAT1, and p-STAT3/STAT3 in miR-135a and AG490 groups were significantly lower than those in the OVA group (P<0.05). p-JAK3/JAK3, p-STAT1/STAT1, and p-STAT3/STAT3 in the miR+BDNF group were significantly higher than those in miR-135a group (P<0.05).

## Discussion

miRNA is a kind of short non-coding RNA with a length of about 22-25 nucleotides, and it regulates gene expression from post-transcriptional levels by degrading or inhibiting translation of target mRNAs (15,21). Asthma is a genetically susceptible chronic airway inflammatory disease involving a variety of cells, cytokines, and signaling pathways that are closely related to immune regulation. A growing body of evidence suggests that miRNAs are involved in the regulation of airway inflammation in asthma (22). Significant differential changes in multiple miRNAs were detected in the lung tissue of the mouse asthma model, and these miRNAs were involved in the regulation of matrix metalloproteinases, inflammatory responses, transforming growth factor beta signaling, and some signaling pathways including apoptosis and inflammation (23,24).

The current study found that miRNAs play an important role in asthma, and can also be used for the susceptibility judgment of asthma genes. Some miRNAs can inhibit the phenotype of asthma and reduce the helper T cell (Th) 2 response, airway hyperresponsiveness, eosinophil accumulation, and weakening airway mucus secretion, thereby inhibiting the inflammatory response of asthma (25,26). A single miRNA can bind to hundreds of target genes and participate in the regulation of inflammatory diseases and immune-related diseases through post-transcriptional regulation of target genes. It is an important regulator of negative feedback regulation (27). miRNAs can regulate the immune system involved in the inflammatory process of asthma in a variety of ways. Th1/Th2 imbalance is one of the important factors in the pathogenesis of asthma, and many miRNAs play an important role in regulating Th1/Th2 (28,29). miRNA is not only a regulator of asthma pathogenesis, it is also an important target for asthma treatment. Some studies have shown that up-regulated miR-135a can inhibit the proliferation of airway smooth muscle cells, which was known to be involved in the pathophysiology of asthma (30).

JAK/STAT signaling pathway is one of the important pathways of cytokine signaling. It is not only involved in inflammatory response, but also closely related to proliferation, differentiation, apoptosis, and immune regulation. This pathway is composed of the tyrosine kinase JAK family and the transcription factor STAT family. The JAKs family is a non-receptor tyrosine protein kinase with a molecular weight of about 120-140 kDa. So far, the JAK protein family has been found to have four members, namely JAK1, JAK2, JAK3, and TYK2. The JAK1, JAK2, and TYK2 can be found in almost all cells and tissues, while JAK3 is mainly found in the lymphatic system and bone marrow (31). The STAT family is a class of target proteins downstream of JAKs, and six members of the STAT family, STAT1 to STAT6, have been discovered. The conduction process is as follows: cytokines such as IL-1 and IL-6 bind to their receptors and cause dimerization of the receptor molecule, which enables JAKs to be close to each other and activated by reciprocal tyrosine phosphorylation. The STAT family is an important component of JAK/STAT signaling pathway, which is especially important in the occurrence and development of asthma (32). Silencing the STAT1 gene in airway epithelial cells of asthmatic mice reduces the number of eosinophils and IL-5 in lung tissue of asthmatic mice, reducing airway inflammation. Studies have also shown that STAT5 gene in T lymphocytes of asthmatic rats can inhibit the proliferation and spread of T lymphocytes, promote autophagy of T lymphocytes, and thus reduce airway inflammation in asthmatic rats (33). Our findings are consistent with those above.

This research used OVA to establish an asthma model to detect airway hyperresponsiveness and further detect the expression of JAK/STAT signaling pathway-related proteins in the later stage. The results of the experiments indicated that miR-135a agonist can inhibit airway inflammation in asthmatic mice, and its mechanism was related to the regulation of JAK/STAT signaling pathway. However, the specific mechanism of action is unclear, and there are many downstream target genes in this signaling pathway. Specifically, which genes play a role in this signaling pathway needs further research.
